# Screening for Potential Therapeutic Agents for Non-Small Cell Lung Cancer by Targeting Ferroptosis

**DOI:** 10.3389/fmolb.2022.917602

**Published:** 2022-07-14

**Authors:** Xin Zhao, Lijuan Cui, Yushan Zhang, Chao Guo, Lijiao Deng, Zhitong Wen, Zhihong Lu, Xiaoyuan Shi, Haojie Xing, Yunfeng Liu, Yi Zhang

**Affiliations:** ^1^ Department of Pharmacology, Shanxi Medical University, Taiyuan, China; ^2^ Department of Endocrinology, First Hospital of Shanxi Medical University, Shanxi Medical University, Taiyuan, China

**Keywords:** ferroptosis, prognostic, non-small cell lung cancer, CMap database, antitumor drug

## Abstract

Ferroptosis is a form of non-apoptotic and iron-dependent cell death originally identified in cancer cells. Recently, emerging evidence showed that ferroptosis-targeting therapy could be a novel promising anti-tumour treatment. However, systematic analyses of ferroptosis-related genes for the prognosis of non-small cell lung cancer (NSCLC) and the development of antitumor drugs exploiting the ferroptosis process remain rare. This study aimed to identify genes related to ferroptosis and NSCLC and to initially screen lead compounds that induce ferroptosis in tumor cells. We downloaded mRNA expression profiles and NSCLC clinical data from The Cancer Genome Atlas database to explore the prognostic role of ferroptosis-related genes. Four prognosis-associated ferroptosis-related genes were screened using univariate Cox regression analysis and the lasso Cox regression analysis, which could divide patients with NSCLC into high- and low-risk groups. Then, based on differentially expressed risk- and ferroptosis-related genes, the negatively correlated lead compound flufenamic acid (FFA) was screened through the Connective Map database. This project confirmed that FFA induced ferroptosis in A549 cells and inhibited growth and migration in a dose-dependent manner through CCK-8, scratch, and immunofluorescence assays. In conclusion, targeting ferroptosis might be a therapeutic alternative for NSCLC.

## Introduction

Lung cancer is one of the most common malignant tumors in China and the world. Non-small cell lung cancer (NSCLC) accounts for 85% of all lung cancers and is the main cause of cancer-related deaths worldwide (Liu et al., 2017). NSCLC, including adenocarcinoma, squamous cell carcinoma, and large cell carcinoma, is a heterogeneous group of diseases that is frequently diagnosed at advanced or metastatic stages (Best and Sutherland, 2018; La Montagna et al., 2020). It is well known that various prognostic factors based on clinical and pathological characteristics determine the overall prognosis of patients, and the most important prognostic factors remain the stage and presentation of the disease at the time of diagnosis (Gadgeel and Thakur, 2016). The lack of obvious symptoms in the early stages of disease progression and late diagnosis might explain the observed poor prognosis (Liu et al., 2017). Han K and Wang Y et al. demonstrated that ferroptosis-related genes are closely associated with the prognosis of NSCLC patients, which will allow for the effective treatment of patients (Han et al., 2021; Wang et al., 2022).

Ferroptosis is an iron-dependent, non-apoptotic mode of programmed cell death that is driven by the lethal accumulation of lipid peroxides (Liang et al., 2020; Li Y. et al., 2020). Previous studies have reported that ferroptosis plays a vital role in NSCLC, and certain genes, such as SLC7A11 (Ji et al., 2018; Kang et al., 2019) and GPX4 (Lai et al., 2019), are known to negatively regulate ferroptosis; moreover, NFS1, a ferroptosis-related gene, is most highly expressed in well-differentiated lung adenocarcinomas and protect cells from ferroptosis (Alvarez et al., 2017). The activation of ferroptosis by several small molecules and FDA-approved clinical drugs in cancer cells and the efficacy of tumor suppression with ferroptosis inducers in various experimental cancer models underline the potential of ferroptosis to be targeted as a novel anti-cancer therapy (Stockwell et al., 2017). Therefore, this study intended to screen out new lead compounds for the treatment of NSCLC based on the induction of ferroptosis in NSCLC cells using bioinformatics theory.

In this study, we used mRNA expression data from NSCLC patients in The Cancer Genome Atlas (TCGA) database to construct a prognostic multigene signature with ferroptosis-related differentially expressed genes (DEGs). These ferroptosis-related risk signatures could independently and effectively classify patients with NSCLC with a high risk of unfavorable outcomes. Finally, we preliminarily screened and proved that the lead compound, flufenamic acid, could induce ferroptosis with anticancer effects by using biological experiments and bioinformatics analysis. These results provide a new strategy for the treatment of NSCLC, help to reveal the association between ferroptosis-related genes and patient prognosis, and also offer basic research data for targeting ferroptosis in the treatment of NSCLC.

### Materials and Methods

#### Reagents

The Cell Counting Kit-8 (CCK-8, C0005) and flufenamic acid (FFA, T0858) were purchased from Taoshu Biotechnology Co., Ltd. Ferrostatin-1(Fer-1, HY-100579) was purchased from MedChemExpress Company. Rabbit anti-human glutathione peroxidase 4 (GPX4, A1933) monoclonal antibody was received from ABclonal Technology Co.,Ltd. The Goat Anti-Rabbit lgG H&L/FITC antibody (bs-0295G-FITC) was acquired from Beijing Boaosen Biotechnology Co., Ltd. Glutathione (GSH, A126-1-1) detection kits was purchased from Nanjing Jiancheng Bioengineering Institute.

#### Downloading mRNA Expression Profiles and Clinical Information

The RNA sequencing data of HTSeq-FPKM and relevant clinical information from the NSCLC cohort were downloaded from TCGA database (https://portal.gdc.cancer.gov/repository). RNA-seq data and clinical information from 228 tumor samples were obtained from the GEO database (GSE 37745, GSE102287, https://www.ncbi.nlm.nih.gov/geo).

#### Extraction of Ferroptosis-Related Genes From TCGA and GEO Databases

60 ferroptosis-related genes were retrieved from previous literature (Stockwell et al., 2017; Bersuker et al., 2019; Hassannia et al., 2019) and are provided in Supplementary Table S1. The R package “limma” and “sva” were used to extract mRNA expression levels of ferroptosis-related genes in TCGA and GEO databases.

#### Identification of Differentially Expressed Ferroptosis-Related Genes in the TCGA Cohort

DEGs related to ferroptosis were screened using the R package “limma” (Ritchie et al., 2015). The expression of candidate ferroptosis-related genes in the TCGA cohort was used to identify the DEGs between tumor tissues and adjacent nontumorous tissues. The screening criteria were set as a *p*-value < 0.05 and false discovery rate (FDR) < 0.05.

#### Identification of Prognosis-Associated Ferroptosis-Related Genes in TCGA Cohort

Univariate Cox analysis regression was performed to evaluate the association between DGEs and patient survival time in TCGA cohort using the R package “survival”, and to obtain the corresponding *p*-value and hazard ratio (HR) for each differential gene. When the *p*-value was <0.05, it was considered that the ferroptosis-related gene was connected with patient prognosis. The higher the HR, the higher the risk of the disease.

#### Construction of a Multigene Signature by Lasso Cox Regression Analysis

The R package “glmnet” was used to perform lasso regression analysis. The candidate ferroptosis-related genes were subjected to univariate Cox linear regression analysis using the R package “survival”. The candidate ferroptosis-related gene was taken as the included signature variable, and the variables were added or deleted until the optimum multigene signature to evaluate the overall survival (OS) outcome was found. The formula for the risk score was as follows: risk score = the sum of each coefficient of mRNA multiple for the expression of each mRNA.

#### Survival Analysis Based on the Stratification of Low- and High-Risk Scores

Patients were divided into high and low-risk groups based on the median risk score of TCGA cohort. The R package “survival” and “survminer” was uesd to perform Kaplan–Meier survival analysis and log-rank tests, which were used to evaluate the OS difference between high- and low-risk groups. A *p-*value of the log-rank test was less than 0.05 indicated a statistically significant difference in survival between the two groups. To validate the survival analysis of TCGA cohort, the Kaplan-Meier method was performed on the GEO cohort to evaluate the OS difference between high- and low-risk groups.

#### Validation of Risk Score by Univariate and Multivariate Cox Analyses

The patient risk scores and clinicopathological characteristics such as age, sex, tumor stage, and grade were taken as variables, and the corresponding survival status was taken as dependent variables. Univariate and multivariate Cox regression analyses were conducted to obtain the risk ratio HR and seek factors that could independently be used to evaluate a patient’s risk of developing the disease. A factor was used as an independent risk factor when the *p*-value was <0.05.

#### Validation of Risk Score by Generating Receiver Operating Characteristic Curve

The R package “survivalROC” was used to generate a receiver operating characteristic curve (ROC curve), which was used to evaluate the specificity and sensitivity of the risk score for patient survival prediction and the diagnostic score for the prediction of patient diagnosis. The robustness of the risk score was evaluated by comparing the area under the curve (AUC) of the ROC curves for different factors.

#### Preliminary Screening of Therapeutic Drugs for NSCLC Using the Connective Map Database

The differential genes related to ferroptosis and those of high- and low-risk patients were entered into the CMap (https://clue.io/query) database in Grp form. With this comparison, related small molecule compounds or drugs were obtained. Taking *p* < 0.05 and enrichment <0 as the screening conditions, the potential small molecule lead compounds for the treatment of NSCLC were screened after the intersection of the two groups of screened drugs.

#### Assessment of Cell Migration and Activity

A549 cells were routinely cultured with 1,640 medium containing 10% fetal bovine serum, digested and passaged, inoculated into a 6-well plate according at 5 × 10^6^ cells/well, or inoculated into a 96-well plate with 1 × 10^4^ cells/well. The cells were cultured in a 5% carbon dioxide incubator for 24 h. After 24 h of drug treatment, the activity in each group of cells was detected using a CCK-8 kit. The cells were incubated in a 96-well plate, 10 μL of CCK-8 solution was added to each well, and the culture was continued at 37°C for 4 h. Absorbance was measured at 450 nm. The formula to calculate cell viability was as follows: cell viability (%) = [A (treatment) − A (blank)]/[A (control) − A (blank)]. The cell wound healing assay was performed according to Li’s research (Li Z.-Y. et al., 2020).

#### Detection of Cellular GSH and GPX4 Levels

A549 cells were divided into a negative control group (NC), FFA-treated group (FFA), and ferrostatin-1 (Fer-1) and combined treatment groups (Fer-1+FFA). After 24 h of drug treatment, the CCK-8 kit was utilized as described. GSH detection kits was used to evaluate the contents of GSH in the cells of each group. The operation steps were performed according to the instructions. Immunofluorescence staining was performed according to Yang’s instructions (Yang et al., 2019). The primary antibody was a rabbit anti-human GPX4 monoclonal antibody, dilution 1:200.

#### Statistical Methods

Spss26.0, Prism 8.0, Image J, SigmaPlot 14, and R software were used for statistical analysis of results (Student’s t-test or one-way ANOVA tests). Data were presented as mean ± standard error of the mean. tje Kaplan-Meier method and log-rank tests were used to evaluate the predictive power of the prognostic model. Univariate Cox regression and multivariate Cox regression were used to identify whether the model could be used as an independent prognostic factor. *p* < 0.05 was considered statistically significant.

## Results

### Profile of the NSCLC Dataset From TCGA and GEO

In this study, 551 cases were obtained from TCGA database, including 54 normal datasets and 497 tumor datasets. A total of 316 cases with clinical characteristics met the inclusion criteria by removing cases with survival periods of less than 30 days with incomplete clinical information. We downloaded a total of 228 cases from the GEO database, including 161 cases with researchable clinical information. The detailed clinical characteristics of these patients, such as age, gender, survival time, and survival status, are summarized in Table 1.

**TABLE 1:** Clinical characteristics of the NSCLC patients used in this study.

Variables	TCGA cohort (*n* = 316)	GEO cohort (*n* = 161)
Age, n (%)		
<65 years	137 (43.35)	88 (54.66)
≥65 years	179 (56.65)	73 (45.34)
Gender, n (%)		
Female	163 (51.58)	88 (54.66)
Male	153 (48.42)	73 (45.34)
Overall survival days, (25%-75%)	596.00 (362.00–947.75)	1366.00 (451.00–2877.00)
Survival status, n (%)		
Alive	203 (64.24)	51 (31.68)
Dead	113 (35.76)	110 (68.32)
Stage, n (%)		
Stage I	164 (51.90)	106 (65.84)
Stage II	76 (24.05)	29 (18.01)
Stage III	56 (17,72)	22 (13.66)
Stage IV	20 (6.33)	4 (2.49)
T, n (%)		
T1	97 (30.70)	NA
T2	178 (56.33)	
T3	24 (7.59)	
T4	17 (5.38)	
M, n (%)		
M0	296 (93.67)	NA
M1	20 (6.33)	
N, n (%)		
N0	200 (63.29)	NA
N1	67 (21.20)	
N2	48 (15.20)	
N3	1 (0.31)	

### Identification of Differentially Expressed Ferroptosis-Related Genes in TCGA Cohort

**FIGURE 1:**
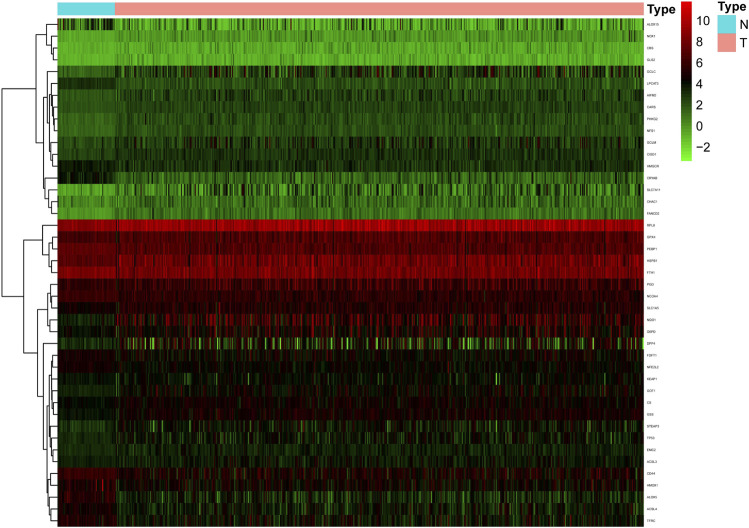
The heatmap of differentially expressed ferroptosis-related genes in the TCGA cohort. Compared with normal samples, a total of 29 up-regulated and 14 down-regulated ferroptosis-related genes were screened in tumor samples.

Sixty ferroptosis-related genes were extracted from TCGA cohort. A nonparametric rank-sum test was performed on 497 patients with tumor tissues and 54 patients with normal lung tissues, and a total of 43 ferroptosis-related genes met the screening criteria of *p*-value < 0.05 and FDR <0.05, including 29 upregulated and 14 downregulated ferroptosis-related genes. These genes were clustered and presented as heatmaps. A heatmap of these differentially expressed ferroptosis-related genes is shown in Figure 1.

### Identification of Prognostic Ferroptosis-Related DEGs in TCGA Cohort

**FIGURE 2:**
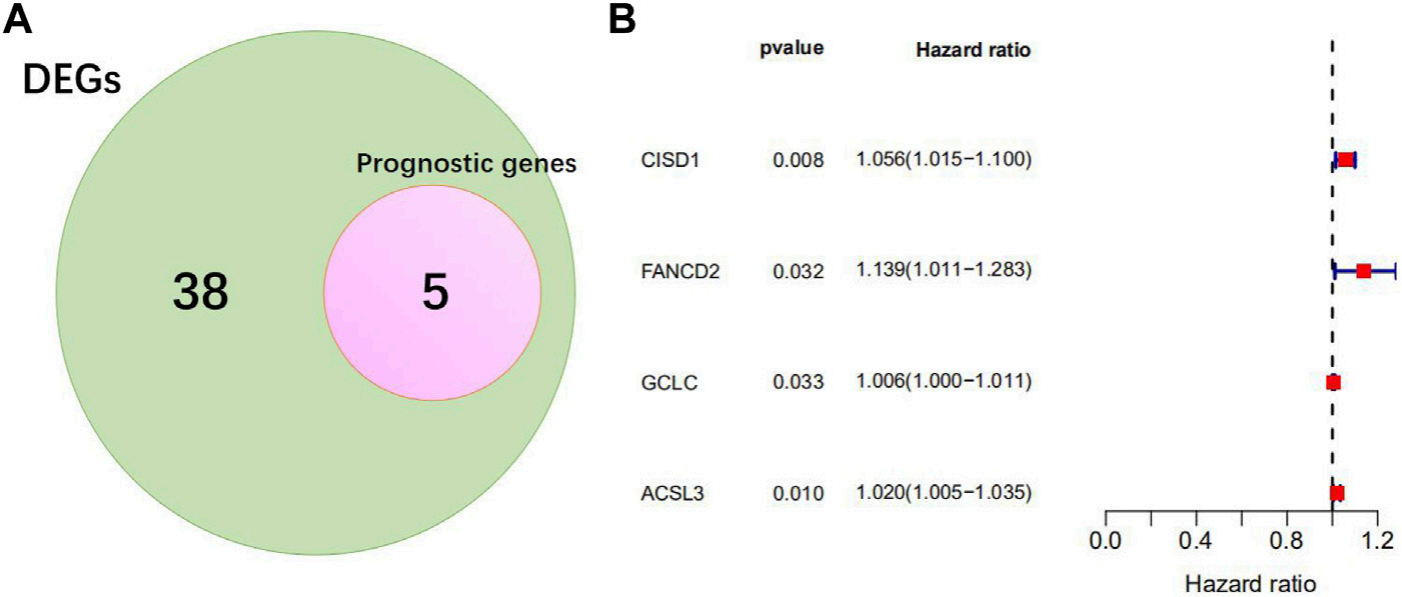
Identification of the candidate ferroptosis-related genes in TCGA cohort. **(A)** Venn diagram to identify differentially expressed genes between tumor and adjacent normal tissue that were correlated with overall survival (OS). The red circle represents the ferroptosis-related genes associated with prognosis, the green circle represents DEGs associated with ferroptosis. **(B)** Forest plots indicating results of the univariate Cox regression analysis between gene expression and OS. *p*-values < 0.05 indicate that ferroptosis-related genes are related to the prognosis of NSCLC. When the hazard ratio (HR) is >1, the gene is considered a high-risk gene.

In view of the above differentially expressed ferroptosis-related genes, combined with the clinical information on survival status and OS days patients in the TCGA cohort, five ferroptosis-related genes related to the prognosis of NSCLC were preliminarily screened using univariate Cox analysis (Figure 2A). Irrationally, *PHKG2* was excluded from further studies, as its expression in univariate Cox regression analysis predicted it to be a low-risk gene with an excellent prognosis. Four prognostic ferroptosis-related DEGs were identified as key genes using lasso regression analysis and preserved (Figure 2B).

### Construction of a Risk Score in TCGA Cohort

Based on the four ferroptosis-related genes identified using lasso regression analysis, the prognostic risk score of each patient was calculated using the risk score expression described above. The risk score = 0.0415124361172914 × expression of *CISD1* + 0.0643218824479954 × expression of *FANCD2* + 0.0018714013929312 × expression of *GCLC +* 0.0139528364527794 × expression of *ACSL3*. All patients were divided into high-risk and low-risk groups according to the median risk score of TCGA cohort.

### Validation of Survival Analysis of TCGA Cohort by Utilizing Data From the GEO Cohort

**FIGURE 3:**
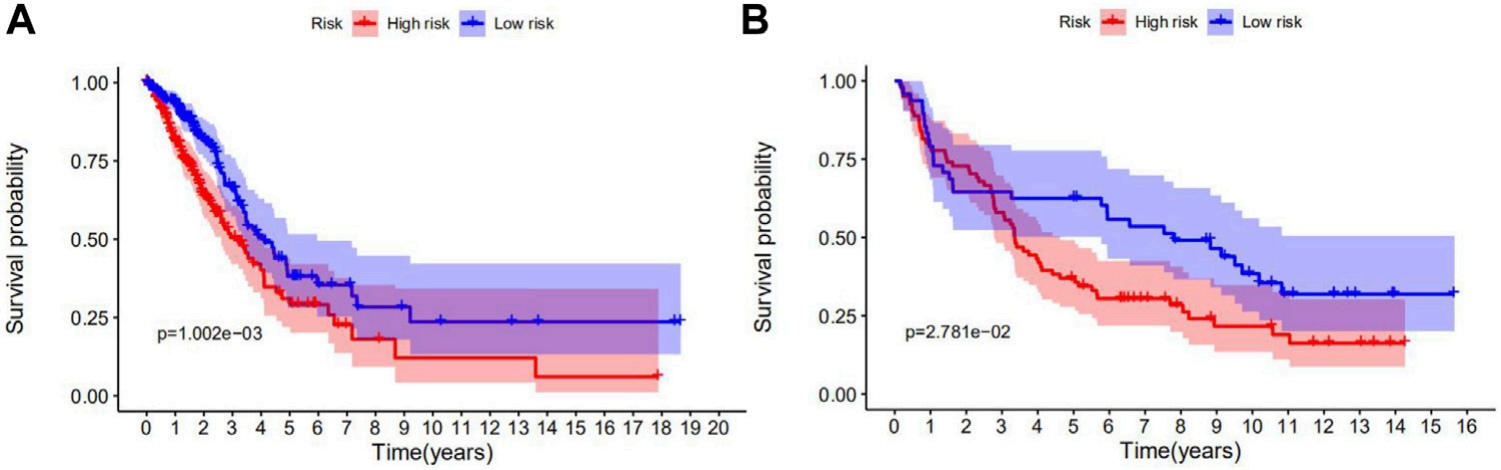
Survival analyses of the prognostic ferroptosis-related genes in NSCLC using the Kaplan-Meier method. **(A)** The Kaplan-Meier curve of TCGA cohort. **(B)** The Kaplan-Meier curve of the GEO cohort. *p*-values < 0.05 indicate that the OS differed significantly between high- and low-risk patients.

Kaplan-Meier survival analysis showed that the OS of the low-risk group was higher than that of the high-risk group in both TCGA (Figure 3A) and GEO cohorts (Figure 3B). *p*-values < 0.05 indicate that the difference in OS curves between high-risk and low-risk groups is considered statistically significant.

### Validation of Risk Score, Survival Status Distribution, and Heatmap of TCGA Cohort Utilizing Data From the GEO Cohort

**FIGURE 4:**
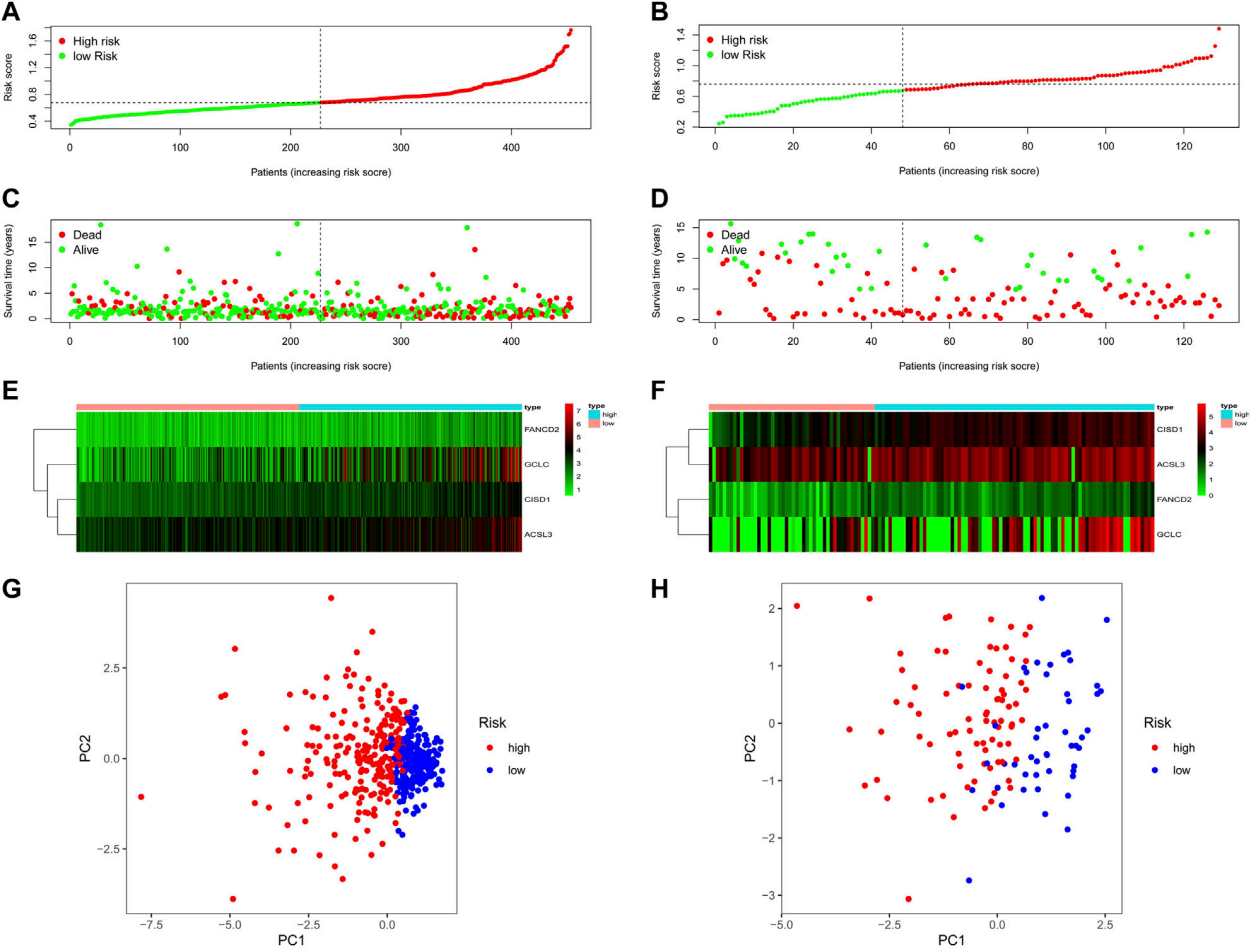
The risk score, survival status distribution, heatmap and PCA plot of TCGA and GEO cohort. **(A)** The distribution and median value of the risk scores in TCGA cohort. **(B)** The distribution and median value of the risk scores in the GEO cohort. **(C)** The distributions of OS status, OS, and risk score in TCGA cohort. **(D)** The distributions of OS status, OS, and risk score in the GEO cohort. **(E)** The heatmap for prognosis-associated ferroptosis-related genes in TCGA cohort. **(F)** The heatmap for prognosis-associated ferroptosis-related genes in the GEO cohort. **(G)** PCA plot of TCGA cohort. **(H)** PCA plot of the GEO cohort.

The patients were separated into high- or low-risk groups based on the median cut-off value (Figures 4A,B). In both TCGA and GEO cohorts, the risk score increased from left to right. The distribution of the survival status demonstrated that the number of deaths increased gradually with the increase in risk value. High-risk patients displayed shorter OS days and a higher probability of early death compared to low-risk patients (Figures 4C,D). Further, the heat maps clearly indicated that the expression of *CISD1, FANCD2, GCLC,* and *ACSL3* were added in the wake of the increase in risk value in both TCGA and GEO cohorts (Figures 4E,F). Based on the expression of this four potential risk genes, we classified patients with NSCLC into high- and low-risk groups. PCA analysis clearly indicated that patients in different risk groups were distributed in two directions (Figures 4G,H).

### Univariate and Multivariate Cox Analysis

**FIGURE 5:**
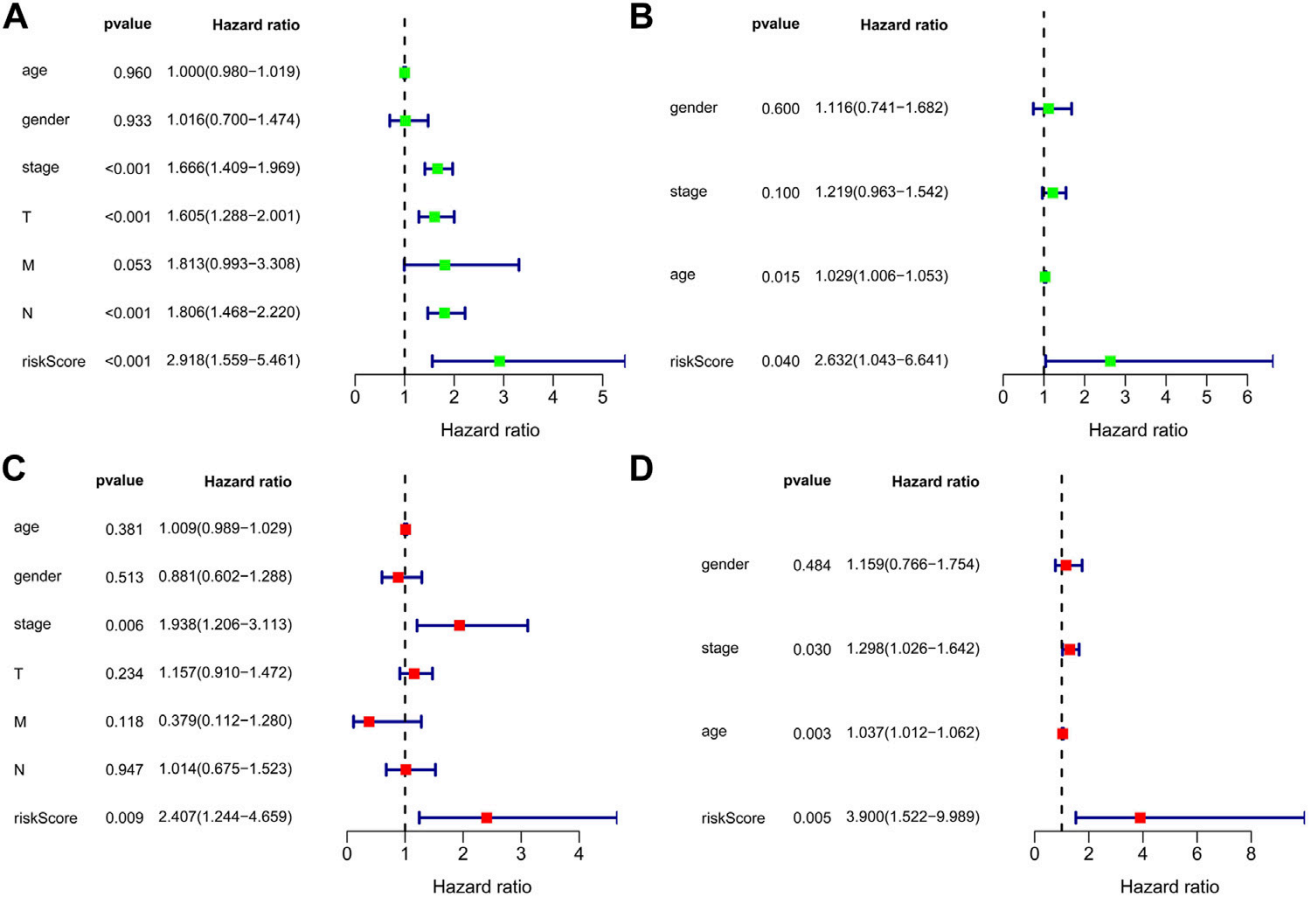
Forest plot of the univariate and multivariate Cox regression analyses in NSCLC. Forest plot of the univariate Cox regression analysis of TCGA cohort **(A)** and GEO cohort **(B)**. Forest plot of the multivariate Cox regression analysis of TCGA cohort **(C)** and GEO cohort **(D)**.

Univariate and multivariate Cox regression analyses were used to examine whether the predicted risk incidence of the risk score was independent of clinical data in both TCGA and GEO cohorts. The risk score was significantly correlated with OS in both TCGA and GEO cohorts in univariate Cox regression analyses (HR = 2.918, *p* < 0.001; HR = 2.632, *p* = 0.040, respectively). Surprisingly, in TCGA cohort, stage is considered interrelated to OS, but cannot be verified in the GEO cohort (Figures 5A,B). The risk of poor survival outcomes increased with an increase in the risk score. After adjusting for other intricate factors, multivariate Cox regression analysis demonstrated that the risk score remained an independent predictor of OS in NSCLC patients (HR = 2.407, *p* = 0.009; HR = 3.900, *p* = 0.005, respectively, Figures 5C,D.)

### Receiver Operating Characteristic Curve Analysis

**FIGURE 6:**
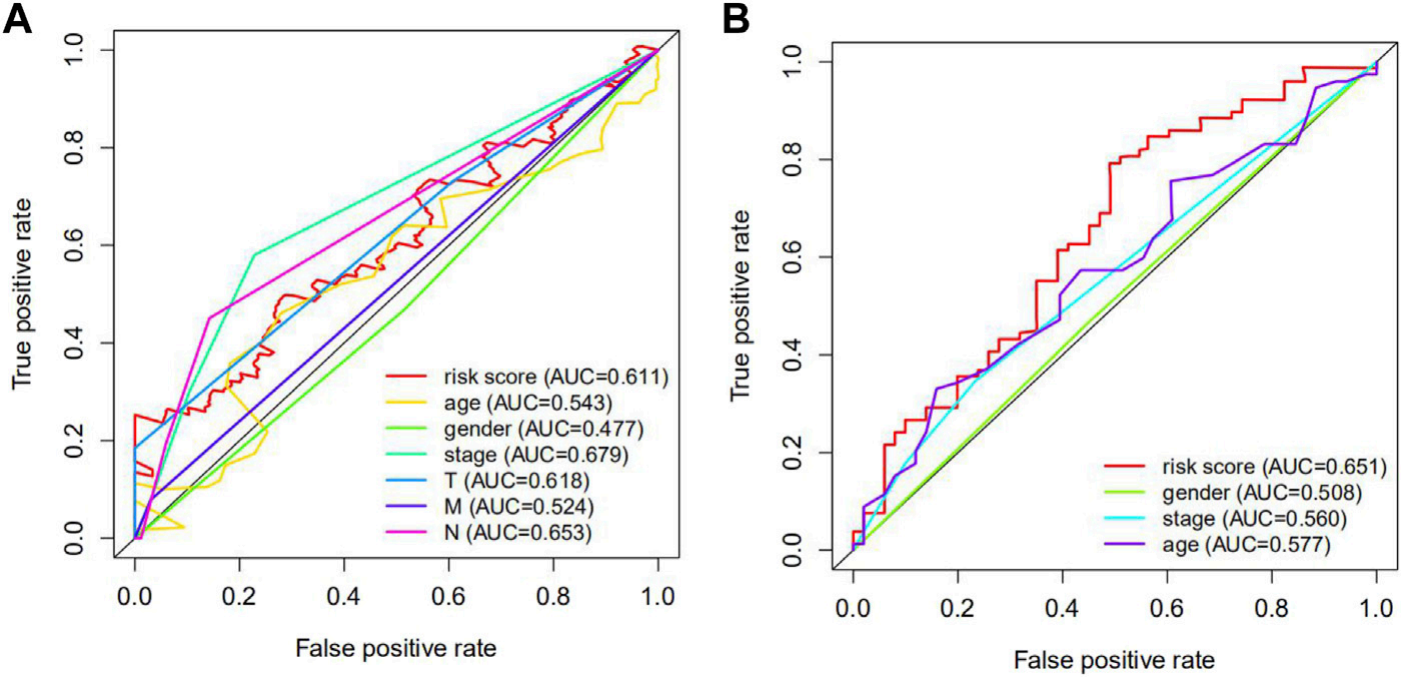
The receiver operating characteristic (ROC) curve of TCGA and GEO cohorts. **(A)** the ROC curve of TCGA cohorts. **(B)** the ROC curve of GEO cohorts. The AUC of the ROC curve was more than 0.5, which signified that the predictive model possessed extraordinary diagnosis and prediction values.

Receiver operating characteristic (ROC) analysis, which is a method combining sensitivity and specificity, was performed to comprehensively evaluate the multivariate Cox regression analysis for long-term survival prediction (7 years). The area under the curve (AUC) of the risk score in TCGA cohorts was 0.611 (Figure 6A) and the area under the curve (AUC) of the risk score in GEO cohorts was 0.651 (Figure 6B), making it clear that the predictive model possessed powerful prognostic capability in forecasting overall survival.

### Screening Potential Therapeutic Agents to Induce Ferroptosis in High-Risk Groups Using the CMap Database

**FIGURE 7:**
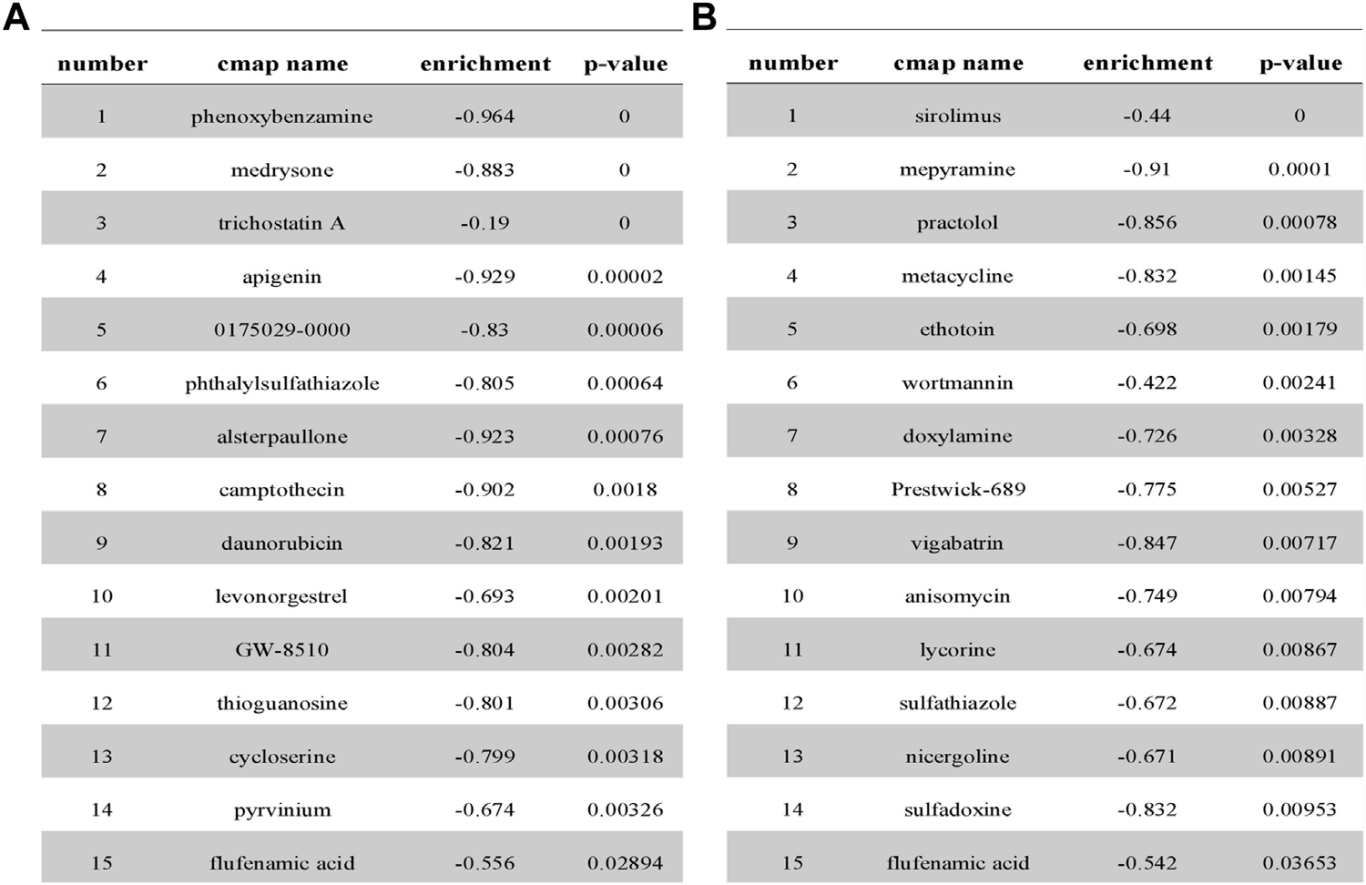
Partial analysis results from CMap database. **(A)** Prediction results from cMap for the differential gene profiles of high- and low-risk groups. **(B)** Prediction results from cMap for the differential gene profiles of DEGs related to ferroptosis.

Based on the query results of the CMap database, the partial potential lead compounds for the treatment of high-risk NSCLC patients are listed in Figure 7A, and the partial potential lead compounds that might trigger ferroptosis in NSCLC are listed in Figure 7B. After the intersection of the two groups of screened drugs, we believed the small molecule compound, FFA, was a therapeutic agent to induce ferroptosis in NSCLC. Previous studies have found that FFA exerts antitumor effects (Matsumoto et al., 2016; Li Z.-Y. et al., 2020), suggesting that our screening strategy and results possess preferable credibility.

### FFA Decreases the Viability and Migration of A549 Cells

**FIGURE 8:**
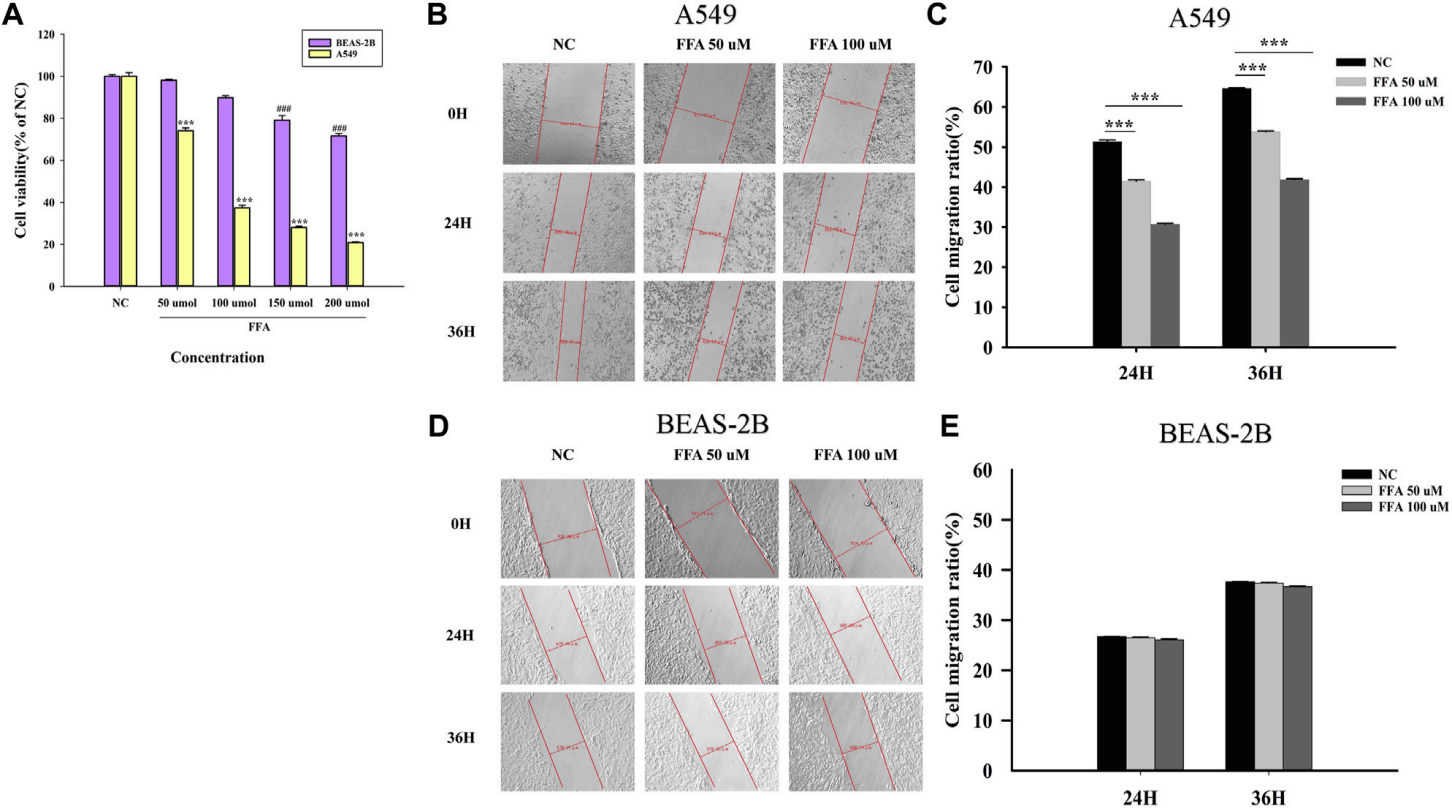
Flufenamic acid inhibits cell viability and migration in A549 cells. **(A)** Effects of different concentrations of flufenamic acid on the cellular activity in A549 cells and BEAS-2B cells (*n* = 5).***p < 0.001 vs. NC of A549 cells. ^###^ *p* < 0.001 vs. NC of BEAS-2B cells. **(B)** The scratch healing of A549 cells in different time periods. The scale bar is 50 μm. **(C)** Statistical chart of cell migration ratio in A549 cells (*n* = 3). ***p < 0.001 vs. NC. **(D)** The scratch healing of BEAS-2B cells in different time periods. The scale bar is 50 μm. **(E)** Statistical chart of cell migration ratio in BEAS-2B cells (*n* = 3). NC, negative control group.

After exposing cells to different concentrations of FFA (50, 100, 150, 200 μmol/L) for 24 h, as shown in Figure 8A, cell viability was reduced with an increase in the FFA concentration. Cell migration into the wound was measured according to the distance between the wound edges before and after FFA treatment. We found that FFA significantly impaired cell migration into the wound (Figures 8B,C). As shown in Figures 8A,D,E, low doses of FFA (50 and 100 μmol/L) had a negligible effect on the growth of normal lung cells (BEAS-2B).

### FFA Partially Induces Ferroptosis in A549 Cells by Repressing the GSH-dependent GPX4 Signaling Pathway

**FIGURE 9:**
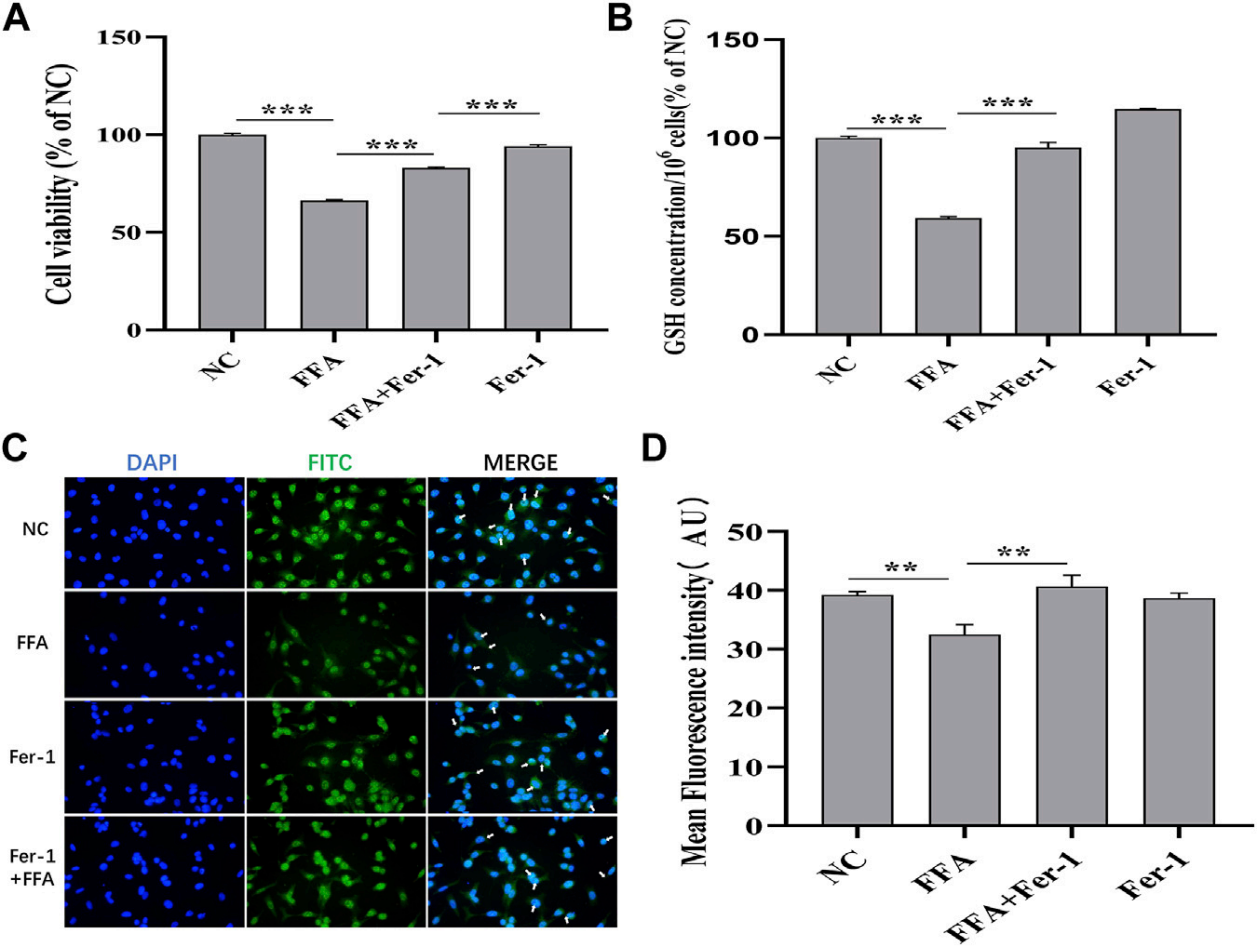
Flufenamic acid(FFA) partially induces ferroptosis in A549 cells by repressing GSH-dependent GPX4 signaling pathway. **(A)** Effect of Ferrostatin-1(Fer-1) on flufenamic acid-restrained cellular activity in A549 cells (*n* = 5). **(B)** Fer-1 reverses the reduced levels of GSH by FFA in A549 cells (*n* = 3). **(C)** Observation of GPX4 immunofluorescence staining in each group (×400). **(D)** Statistical diagram of the average fluorescence intensity of cells in each group (*n* = 6). NC, negative control group. **p < 0.01 and ***p < 0.001.

It is well known that the GSH depletion caused by cysteine deficiency directly inactivates glutathione peroxidase 4 (GPX4) and leads to subsequent ferroptosis (He et al., 2022). Our results showed that FFA remarkably reduced the expression of GSH and GPX4 in A549 cells, whereas Fer-1, an inhibitor of ferroptosis, significantly reversed this trend (Figures 9B–D). The A549 cell activity reduced by FFA could not completely reversed by Fer-1, indicating that the antitumor effect of FFA was not only mediated by ferroptosis (Figure 9A). Therefore, we concluded that FFA partially induced ferroptosis in NSCLC cells by repressing GSH-dependent GPX4 signaling.

## Discussion

### Ferroptosis-Related Genes Could Be Used as Prognostic Markers of NSCLC

Lung cancer is one of the most common malignancies and accounts for nearly one quarter of cancer deaths worldwide (Siegel et al., 2021). The most representative histological type of lung cancer is NSCLC, which is generally diagnosed at an advanced stage owing to finite symptoms at the early stage and the restriction of proteome biomarkers (Wu et al., 2021). Patients with lung cancer generally experience tumor recurrence and metastasis, leading to a comparatively poor OS rate (Zhou et al., 2020). Therefore, there is an urgent need to conduct more in-depth research on the identification of novel diagnostic or prognostic markers and potential drug targets to promote prognosis and personalized treatment. With the innovative development of gene chips and next-generation sequencing technology (NGS), gene signatures based on abnormal mRNA have shown the ability to predict the OS outcomes for malignant tumors (Njoku et al., 2020; Tan et al., 2020). Utilizing genomics and/or transcriptome analysis of neoplasm biopsy samples to infer disease severity is relatively fast and inexpensive. Doctors can use NGS to analyze multiple genes associated with an increased cancer risk at one time (Judkins et al., 2015; Fazio et al., 2020). According to NGS, doctors can accurately obtain the genetic variation information of patients, select potential targeted drugs for patients, evaluate the prognosis of patients, design drug-resistance treatment regimens, and realize individualized treatment (Van Allen et al., 2014; Hagemann et al., 2015; Forschner et al., 2021; Grobbel et al., 2021). In recent years, ferroptosis has attracted much attention because it plays a significant role in the occurrence, development, and multidrug resistance of tumors (Liang et al., 2019; Xia et al., 2019). Convincing evidence suggests that ferroptosis can inhibit tumorigenesis (Mou et al., 2019). Moreover, the genes associated with ferroptosis can be used as candidate biomarkers for tumor therapy (Zhuo et al., 2020). Hence, based on RNA sequencing data from TCGA and GEO, we evaluated the relationship between the expression profile of ferroptosis-related genes and the prognosis of patients with NSCLC. In this study, more than two-thirds of ferroptosis-related genes were differentially expressed between tumor and adjacent normal tissues, indicating that there is an association between ferroptosis and NSCLC. Four prognosis-associated ferroptosis-related genes (*FANCD2*, *ACSL3*, *GCLC*, and *CISD1*) were identified using univariate Cox regression analysis and a lasso Cox regression analysis. Our study indicated that the OS outcome was worse for patients with high risk score (*p* < 0.05) in Kaplan-Meier analysis. Analyses such as survival status distribution and PCA showed that the prognostic multigene signature notably divided high- and low-risk patients into two categories. In brief, a prognostic multigene signature could not only be used for risk assessment and the identification of high-risk patients but also to ameliorate nursing care by changing treatment schemes (Judkins et al., 2015), promote individualized treatment, and improve cancer treatment efficiency.

### Prognosis-Associated Ferroptosis-Related Genes Provide Opportunities for Neoplastic Therapeutic Approaches

These four prognosis-associated ferroptosis-related genes were up-regulated in NSCLC cancerous tissues and associated with poor prognosis, which are also different from the findings of Han et al. (Lai et al., 2019). Our results further broadened the prognosis-associated ferroptosis-related gene signatures for NSCLC. *FANCD2* overexpression increases the risk of metastasis in esophageal squamous cell carcinoma (ESCC) by activating DNA replication and regulating cell cycle progression, which is associated with the negative prognosis of ESCC (Lei et al., 2020). Bone marrow stromal cells (BMSCs) lacking *FANCD2* exhibit iron overload and lipid peroxidation with erastin (inducing ferroptosis to selectively kill NSCLC cells) (Song et al., 2016; Gai et al., 2020). Monounsaturated fatty acids activated by *ACSL3* can protect cells against ferroptosis (Magtanong et al., 2019). High levels of *ACSL3* are frequently positively associated with poor clinical outcomes in patients with advanced NSCLC (Fernández et al., 2020). High mRNA expression of *GCLC* in tumor tissue significantly shortens the postoperative recurrence survival period of patients, and potentially can predict cisplatin resistance in patients with lung adenocarcinoma (Hiyama et al., 2018). *GCLC* promotes the ferroptosis-resistant state of NSCLC cells by preserving the glutamate balance (Kang et al., 2021). *CISD1*, also known as mitoNEET, is instrumental for the proliferation, migration, and invasion of tumor cells and accelerates the occurrence of malignant tumors (Mittler et al., 2019). Genetic inhibition of *CISD1* induces iron-mediated lipid peroxidation in the mitochondria, which intensifies the strength of erastin-induced ferroptosis (Yuan et al., 2016). Unfortunately, whether *CISD1* has a marked effect on the prognosis of malignant tumors has not been reported. Based on these results, we speculated whether the high-risk ferroptosis-related genes could be genetically suppressed to induce NSCLC cell death. We went a step further by investigating the lead compound with potential therapeutic effects in NSCLC.

### FFA Suppresses Tumor Development by Partially Inducing Ferroptosis in NSCLC Cells

At present, the research and development (R&D) costs of new molecular entity drugs have increased significantly, and the R&D cycle appears to be lengthy (Yildirim et al., 2016). Drug repurposing, that is, a novel drug development strategy to discover new drug indications based on drugs already on the market or in the clinical research stage, can mitigate a variety of costs including time, manpower, and material resources (Parvathaneni et al., 2019). Application of the CMap database can reveal drug–gene associations and identify possible therapeutic effects of compounds (Shi et al., 2016; Li H. et al., 2020). Based on gene chip technology and the CMap database for drug repositioning, we identified the potential candidate lead compound FFA to induce ferroptosis in NSCLC cells and cure this disease. We then used biological techniques to investigate whether FFA plays an important role in inhibiting the growth and metastasis of NSCLC. As expected, the results of CCK-8 and scratch experiments found that FFA dramatically induced cell death and suppressed proliferation in A549 cells. The small-molecule lead compound FFA is thus expected to be an effective drug for the treatment of NSCLC.

Ferroptosis is a novel mode of regulated cell death mediated by the iron-dependent accumulation of lipid peroxides and lipid reactive oxygen species (ROS). Tumor suppression can be mediated by iron inducers in various experimental cancer models, underscoring the potential of ferroptosis inducers as a new anticancer therapy (Hassannia et al., 2019; Yu et al., 2019; Badgley et al., 2020; Liao et al., 2020). Regulation of the classic ferroptosis-repressed GSH-dependent GPX4 signaling pathway is the dominating mechanism for causing ferroptosis in NSCLC (Shui et al., 2021; Zou et al., 2021). GSH was suggested to be a pivotal factor in maintaining GPX4 activity (Yan et al., 2021). The function of GPX4 is to prevent cells from amassing lipid hydroperoxides and avoid cellular ferroptosis. Studies clearly show that the inhibitor of GPX4 induces ferroptosis in not only cultured cancer cells but also tumor xenografts implanted in mice (Pu et al., 2020; Wang et al., 2020). *In vitro* experiments have shown that ferroptosis can be detected via multifarious methods involving the measurement of cell activity, GPX4, GSH, malondialdehyde, and ROS levels (Hu et al., 2020; Pu et al., 2020; Huang et al., 2022). Our study found that FFA decreased the levels of GSH and GPX4 in A549 cells, which was reversed by Fer-1, confirming that FFA caused ferroptosis in NSCLC cells. However, the A549 cell activity reduced by FFA could not completely reversed by Fer-1, suggesting that FFA suppresses tumor development partially by ferroptosis pathway in NSCLC cells.

Tumor-associated inflammation appears to be a new hallmark of cancer therapy (Li Z.-Y. et al., 2020). It has been widely noticed that FFA is an inhibitor of cyclooxygenase-2 (COX-2) (Pal et al., 2021). COX-2, an important inflammatory factor, is considered a key factor in tumorigenesis and might as a potential marker of poor prognosis in NSCLC (Castelao et al., 2003; Sandler and Dubinett, 2004; Liu et al., 2015). Inhibiting the excessive expression of COX-2 suppresses tumor growth and metastasis (Xu, 2002). Vainio H et al. also concluded that evidence for a cancer-preventive effect of COX-2 inhibitors has also been found in a variety of animal models (Vainio, 2001). Therefore, we speculated that the inhibitory effect of FFA on NSCLC may also be related to the improvement of COX-2-associated inflammation (Gridelli et al., 2002). Regrettably, the role of COX-2 in the treatment of NSCLC with FFA was not investigated in this study. In the future, further experiments will be performed to explore the relationship between COX-2 and FFA-suppressed NSCLC growth. In conclusion, these experimental results confirm the feasibility of targeting ferroptosis for the treatment of NSCLC and further verify that our screening strategy.

## Conclusion

In summary, we analyzed and verified the ferroptosis-related genes for the prognosis of NSCLC, which can be used as a novel biomarker for targeting ferroptosis in the individualized treatment of NSCLC patients. Further, the potential lead compound FFA for the treatment of NSCLC were detected based on the above screening of high-risk ferroptosis-related genes. We also confirmed that FFA induced ferroptosis in A549 cells and inhibited growth and migration in a dose-dependent manner. Our findings also developed a new strategy for the antitumor drugs exploiting the ferroptosis process.

## Data Availability

The original contributions presented in the study are included in the article/Supplementary Material, further inquiries can be directed to the corresponding authors.

## Abbreviations

AUC, area under the curve; BMSCs, bone marrow stromal cells; CCK-8, cell counting kit-8; COX-2, cyclooxygenase-2; CMap database, connective map database; DEGs, differentially expressed genes; ESCC, esophageal squamous cell carcinoma; FFA, flufenamic acid; Fer-1, Ferrostatin-1; GPX4, glutathione peroxidase 4; GSH, Glutathione; HR, hazard ratio; NC, negative control group; NGS, next-generation sequencing technology; NSCLC, non-small cell lung cancer; ROC, receiver operating characteristic; R&D, research and development; ROS, reactive oxygen species; TCGA, the cancer genome atlas.
